# Biosynthesis of Silver Nanoparticles from *Paullinia cupana* Kunth Leaf: Effect of Seasonality and Preparation Method of Aqueous Extracts

**DOI:** 10.3390/ph19010072

**Published:** 2025-12-30

**Authors:** Alan Kelbis Oliveira Lima, Tainá Pereira da Silva Oliveira, Isadora Florêncio, Alberto Gomes Tavares Junior, Victor Hugo Sousa Araújo, Arthur Abinader Vasconcelos, Marlus Chorilli, Hugo de Campos Braga, Dayane Batista Tada, Gerson Nakazato, Sônia Nair Báo, Paulo Sérgio Taube, José Antônio de Aquino Ribeiro, Clenilson Martins Rodrigues, Mônica Pereira Garcia

**Affiliations:** 1 Brazilian Agricultural Research Corporation (EMBRAPA), Embrapa Agroenergy, Brasília 70770-901, DF, Brazil; 2 Nanobiotechnology Laboratory, Institute of Biological Sciences, University of Brasília (UnB), Brasilia 70910-900, DF, Brazil; 3 Department of Genetics and Morphology, Institute of Biological Sciences, Darcy Ribeiro University Campus, University of Brasilia (UnB), Brasília 70910-900, DF, Brazil; 4 Microscopy and Microanalysis Laboratory, Department of Cell Biology, Institute of Biological Sciences, University of Brasília (UnB), Brasília 70910-900, DF, Brazil; 5 School of Pharmaceutical Sciences, São Paulo State University (UNESP), Araraquara 14800-901, SP, Brazil; 6 Structured Nanomaterials Study Group, Federal University of Western Pará (UFOPA), Santarém 68005-120, PA, Brazil; 7 Laboratory of Oils of the Amazon, Institute of Biological Sciences, Federal University of Pará (UFPA), Belém 66075-110, PA, Brazil; 8 Institute of Science and Technology, Federal University of São Paulo (UNIFESP), São José dos Campos 12231-280, SP, Brazil; 9 Basic and Applied Bacteriology Laboratory, State University of Londrina (UEL), Londrina 86057-970, PR, Brazil; 10 Institute of Biodiversity and Forests, Federal University of Western Pará (UFOPA), Santarém 68005-100, PA, Brazil

**Keywords:** nanobiotechnology, metallic nanoparticles, AgNPs, Guarana, Amazon, seasonal variation, extraction methods, bioactive plant compounds

## Abstract

**Background/Objectives**: The biogenic synthesis of silver nanoparticles (AgNPs) is a promising alternative method, driven by the presence of metabolites in plant matrices capable of acting as reducing and stabilizing agents. Seasonality is a key factor that influences the phytochemical composition of plants and can directly impact the yield, physicochemical characteristics, stability, and bioactivities of the obtained AgNPs. This study aimed to synthesize AgNPs using aqueous extracts from *Paullinia cupana* leaves collected during dry and rainy seasons, prepared by two different methods (agitation or infusion), to evaluate the impact of these variables on the biosynthesis and properties of the nanostructures. **Methods**: The extracts were characterized by UHPLC-HRMS/MS, and their total phenolic compound (TPC) content and antioxidant potential against DPPH and ABTS radicals were determined. The AgNPs were characterized by UV/Vis spectrophotometry, dynamic light scattering (DLS), zeta potential (ZP), nano-particle tracking analysis (NTA), transmission electron microscopy (TEM), and energy-dispersive X-ray spectroscopy (EDX). **Results**: The metabolic profile results showed a predominance of alkaloids and flavonoids in all extracts, with greater phytochemical diversity in samples prepared by infusion. TPC indicated superior phenolic extraction in extracts prepared by infusion during the rainy season, correlating with greater antioxidant potential via the elimination of free radicals. The evolution of AgNP synthesis was accompanied by a gradual change in the color of the suspensions and the formation of plasmon bands between 410 and 430 nm, characteristic of spherical AgNPs. The nanostructures presented hydrodynamic diameters between 37.49 and 145.5 nm, PdI between 0.222 and 0.755, and Zeta potential between −11.3 and −39.9 mV, suggesting satisfactory colloidal stability. Morphological analyses revealed predominantly spherical particles with average diameters ranging from 33.61 to 48.86 nm and uniform distribution, while EDX spectra confirmed the presence of silver. **Conclusions**: Thus, our results demonstrate that both seasonality and the method of extract preparation influence the phytochemical composition and, consequently, the morphology, stability, and optical properties of AgNPs, with subtle emphasis on collections made during the rainy season and extracts prepared by infusion. Such knowledge contributes to the advancement of more reproducible and purpose-oriented syntheses in the field of green nanotechnology, enabling applications in various sectors.

## 1. Introduction

The world’s plant biodiversity has emerged as a great field for research in health, either by public or private institutions, where the development of new key areas such as synthetic biology presents an underlying role in the study of plant metabolic pathways being a relevant source of medicinal compounds. It is made possible by diverse approaches, e.g., co-expression analysis, gene cluster identification, metabolite profiling, deep-learning approaches, genome-wide association, and protein studies [1]. One of the bioproducts generated from the bioindustry found especially in the Amazon rainforest is guarana (*Paullinia cupana*), which presents compounds of interest for the food and pharmaceutical industries such as flavonoids and methylxantines (catechins, caffeine, and theobromine) [2,3]. The use of guarana was initially as a tonic by traditional populations, with energetic features due to the presence of methylxanthines. According to the *Brazilian Pharmacopeia*, there are two varieties, sorbilis and typica, belonging to the Sapindaceae family. The main plant part consumed are the seeds, which are separated from the pericarp and crushed to be intake as a fine powder [4,5].

Variations in weather, including seasonality, solar irradiation, water and soil availability, and atmospheric pollution, have great influence on many physiological factors in tropical plants, for instance, on their photosynthetic activity [6,7]. In the Amazon rainforest, seasonality exists with a distinct dry season characterized by less than 100 mm/month that may be in absence in the northwestern Amazon; moreover, in the central eastern equatorial Amazon, the dry season is extended for 5 months. This is an important external factor that influences the chemical composition of plants favored by extreme climatic seasons in the Brazilian Amazon, influencing the bioactive compound profile in crops such as cocoa beans, as well as in terms of the composition of catechin, epicatechin, caffeine, and theobromine [8]. These compounds in guarana seeds are represented by its majority components (methylxanthines), besides saponins, polyphenols, and tannins that were reported to reduce serum levels of cytokines IL-1β, IL-6, and IFN-γ, associated with immuno-response related to inflammation processes [9]. Furthermore, the experimental conditions applied for extraction are relevant, with a highlight on the temperature applied and time of extraction [10].

The production of secondary metabolites is part of an evolutionary feature in plants as a response to environmental changes or stress [11]. An evaluation of external factors influencing secondary metabolite production was made in the *Litchi chinensis* Sonn. plant that is from the same family as guarana. There is a relationship between the species’ flower production and the phenol/flavonoid composition, with an adequate temperature being important to avoid a decrease in plant flower and fruit production, highlighting the importance of these secondary metabolites’ presence for many other plant demands. Phenolic compounds are useful to aggregate substances that may help pollinate, giving colors to avoid the attack of herbivores; moreover, they present antifungal and antibacterial features for the plant. The presence of phenolics is strongly dependent on the environmental conditions and it determines the flavonoid composition that integrates physiological processes of photosensitization, photosynthesis, respiration, and the flavor of plants, one important characteristic in guarana fruits [12].

The influence of dry and rainy periods on the chemical composition of guarana is important due to the possibility of screening the better plant sample collected in different periods to give an adequate medium for biogenic synthesis of metal nanoparticles, which have great potential for biomedical applications as antibacterial agents, for wound healing, and for engineering in production of fuel cells with specific electrocatalytic behavior [13,14,15]. The guarana extract, due to the rich composition of desired organic compounds for synthesis of nanostructures, has been explored for production of other types of structures with potential application in health. Liposomes, biodegradable and biocompatible structures existing as vesicular nanocapsules with amphiphilic characteristics, preserve the properties of encapsulated guarana compounds for any therapeutic purpose [16,17].

The union of studies of varied chemical composition of guarana extracts with the possibility of synthesis of nanoparticle metal may represent a valuable product of nanotechnology development, because of the colloidal formulation formed by phytochemicals extracted from guarana, such as alkaloids and tannins, that together with flavonoids may present anti-inflammatory, antioxidant, and immuno-modulatory activities as observed in periodontal disease. Catechins are other bioactive compounds with properties against oxidative stress, because of their potential as endogenous antioxidants avoiding ischemia and free radical scavenging effects [18,19,20]. The genetic factor has been explored in guarana culture with the formation of a genetic bank that will contribute to new breeding programs with possible control of factors that influence the phytochemical composition [21].

Silver nanoparticle (AgNP) biogenic synthesis represents an eco-friendly approach for a valuable product with previously explored potential biomedical and pharmacological applications [22,23]. Therefore, the aim of this work is to explore the methods of aqueous extraction of bioactive compounds in guarana leaf samples from different climate seasons and explore the potentiality of the extracts to generate AgNPs according to those phytochemical compositions.

## 2. Results and Discussion

### 2.1. Chromatographic Profile of the Metabolite Composition of Aqueous Extracts Characterized by UHPLC-HRMS/MS

Plant metabolism and the presence/concentration of biomolecules can vary considerably depending on biochemical, physiological, ecological, and evolutionary processes, including seasonality, cultivation methods, planting regions, plant age, altitude, and extraction conditions. It is crucial to develop economical, sustainable, and efficient methods for extracting and characterizing phytochemicals from plant sources [24,25].

The results of the analyses of phytochemical constituents by UHPLC-HRMS/MS showed that the composition of the aqueous extracts of *P. cupana* leaves prepared by agitation and infusion was similar, regardless of the time of year when the plant material was collected. The metabolites identified and annotated in the base peak chromatograms (BPCs), as well as details on retention time, mass-to-charge ratio (*m*/*z*), molecular formula, and classes to which they belong, are shown in Table 1.

When evaluating the compounds detected in positive ionization mode ESI(+)-MS (Table 1, Supplementary Figure S1A), the occurrence of proanthocyanidin Cinnamtannin D1 in Ext-I-LD/LR (*m*/*z* 865.1989; tR 11.503–12.155 min) and the flavonoids saponarin (*m*/*z* 595.1667; tR 11.774 min), astilbin (*m*/*z* 451.1241; tR 14.525 min), taxifolin (*m*/*z* 305.0656; tR 14.525 min), and pipecolic acid (*m*/*z* 130.0860; tR 1.548 min) in Ext-I-LD was noted. Among the noted metabolites, the alkaloids fagomine, calistegin B2, and theobromine were the most recurrent, appearing in most extracts with *m*/*z* ratios around 148.09, 176.09, and 181.07, in addition to retention times between 1.072 and 1.112, between 0.986 and 1.023 min, and between 8.155 and 8.697 min, respectively. It is also noteworthy that a greater number of compounds were identified in the extracts prepared by infusion, suggesting that temperature is a determining factor for the extraction of metabolites from *P. cupana* leaves.

In negative ionization mode ESI(−)-MS (Table 1, Supplementary Figure S1B), compounds of the cyclic polyol class such as quinic acid (*m*/*z* 191.0563; tR 1.230 min) were identified in Ext-I-LD, while chalcones (*m*/*z* 449.1087; tR 14.097 min) were detected in Ext-I-LR. However, glycosylated flavonoids were predominant in all analyzed extracts; these were identified as astilbin (*m*/*z* 449.1074–449.1104; tR 13.496–14.289 min), quercitrin (*m*/*z* 447.0917–449.0939; tR 14.320–14.870 min), and afzelin (*m*/*z* 431.0967–431.0983; tR 15.262–15.266 min). Furthermore, using the proposed methodology, it was not possible to detect the presence of any metabolites in Ext-A-LD, and when evaluating the results obtained from this ionization mode, none of the extract preparation methods stood out in terms of the number of extracted compounds.

**Figure 1:**
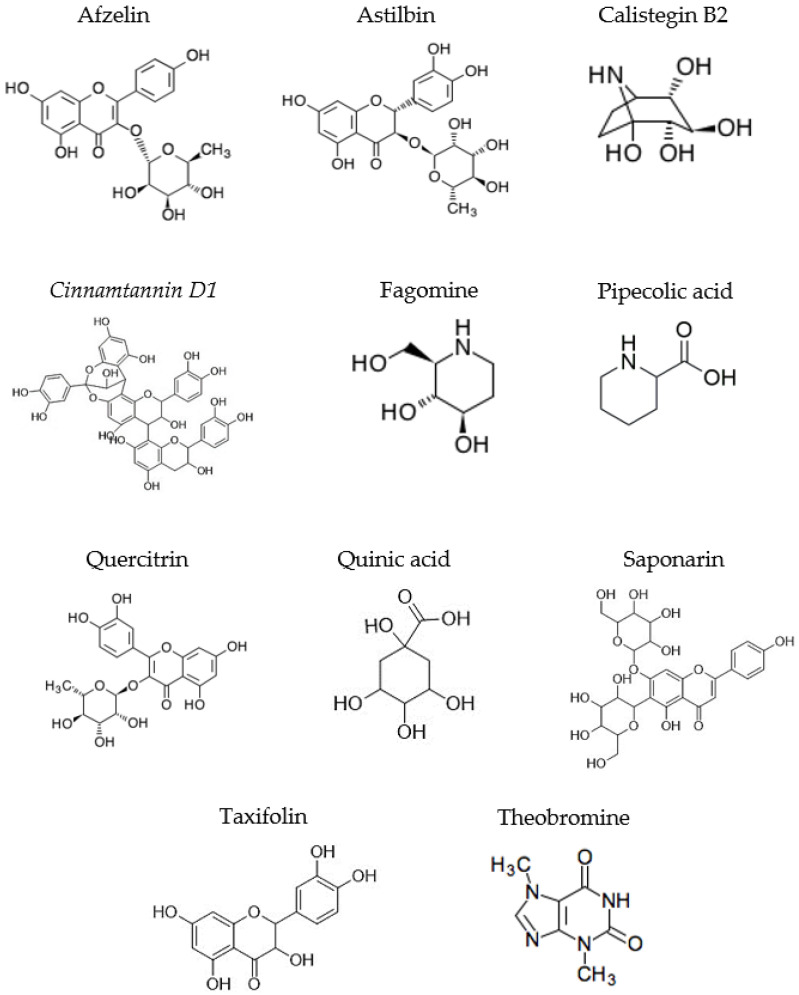
Chemical structures of compounds identified in seasonal aqueous leaf extracts of *Paullinia cupana*.

The phytochemical identification of biomolecules in plant extracts is fundamental to understanding the processes involved in the biogenic synthesis of AgNPs, since these compounds act in the formation of nanostructures. Most of the metabolites detected in this study have functional groups that are suggested to be primarily responsible for the reduction of metal ions, including hydroxyl (-OH), carbonyl (C=O), and amine (-NH-) groups [26] (Figure 1). In fact, the influence of phenolic compounds of plant origin, including flavonoids and alkaloids, on the synthesis of AgNPs is widely recognized and can occur through tautomerization (keto-enol conversion) of flavonoids and the donation of free electrons, in addition to the chelating action of alkaloids when converted to a quinone form under alkaline conditions, thus favoring the reduction of cationic silver ions (Ag^+^) to their colloidal form (Ag^0^) and the stabilization of nanostructures through electrostatic and steric effects [27,28,29,30].

The noted intensity of the peaks generally reflects the relative concentration of biomolecules in the samples, which can affect the physicochemical properties, stability, and bioactivities of the synthesized AgNPs [31,32]. In this context, the occurrence of the alkaloid theobromine and the flavonoid quercitrin in the positive- and negative-mode spectra, respectively, of both forms of extract preparation, presented high peak intensity, indicating probable greater availability in the analyzed aqueous extracts. Corroborating this finding, a recent study also described these biomolecules in aqueous extracts of *P. cupana* flowers from the same geographical region where the leaves in this study were collected [33], demonstrating that the developed analytical methodology can be considered efficient in analyzing the phytochemical composition of guarana leaf extracts, identifying metabolites from different variables.

### 2.2. Quantification of Total Phenol Compounds (TPCs) and Antioxidant Potential

The presence of phenolic compounds is associated with the antioxidant and functional properties of plant extracts, widely recognized for their ability to neutralize free radicals, protect against oxidative stress, and contribute to various biological activities. In this study, based on tests using different forms of preparation of seasonal aqueous leaf extracts of guarana, Table 2 shows the results of TPC and antioxidant potential related to free radical scavenging in each extract sample.

Regarding TPC, the obtained values showed that all extracts differed statistically (*p* < 0.05) from each other. The highest values observed were from extracts of leaves collected during the rainy season, with Ext-I-LR having 281.40 ± 0.04 µg GAE/g and Ext-A-LR having 237.00 ± 0.05 µg GAE/g; among the leaf extracts from the dry season, Ext-I-LD had the highest phenolic content with 203.50 ± 0.06 µg GAE/g, followed by Ext-A-LD with 120.50 ± 0.04 µg GAE/g. Thus, it can be inferred that seasonality and the type of extract preparation directly influence the yield and extraction of total phenolic compounds. In addition to the analyzed variables, in general, this variation in TPC values can be attributed to the extraction solvents, extraction time, and temperature that were used [34].

In previous studies, TPC has been investigated mainly from extracts of *P. cupana* seed powder prepared by different methods, including the use of an ultraturax, which resulted in 104 mg GAE/g after extraction with water for three minutes [35]. In another study, using only ethanol and continuous stirring for 4 h at room temperature, 139 mg GAE/g of phenolic compounds was extracted from the analyzed extract [36]. Extractions using organic solvents demonstrate high efficiency, resulting in a phenolic content of approximately 70, 85, and 90 mg GAE/g when using methanol, ethanol, or acetone, respectively [37], and it was demonstrated that the increase in TPC was proportional to the increase in stirring time from 30 min to 24 h. Still evaluating extracts from guarana seed powder, after 2 h of extraction at room temperature, it was determined that the phenolic composition ranged from 166 mg GAE/g when using a hydroethanolic solution to 181 mg GAE/g with water alone [38]. Although our values are lower, this was expected since we used only water as the extraction solvent, a condition that favors water-soluble compounds.

As shown in Table 2, when evaluating the antioxidant potential of aqueous extracts of *P. cupana* through DPPH elimination, it was noted that the values obtained from the aqueous extracts of leaves from the dry season differed significantly (*p* < 0.05) from each other, with Ext-I-LD registering 34.38 ± 0.05 µg GAE/g and Ext-A-LD registering 23.43 ± 0.06 µg GAE/g. On the other hand, data obtained for leaf extracts from the rainy season showed values close to 29.48 ± 0.04 and 31.11 ± 0.04 µg GAE/g for Ext-A-LR and Ext-I-LR, respectively. Regarding ABTS, the direct influence of the extract preparation method on free radical elimination was observed, since when testing Ext-I-LD and Ext-I-LR, the values were 17.60 ± 0.03 and 17.75 ± 0.02 µg GAE/g, respectively, which were statistically different (*p* < 0.05) from the results found for Ext-A-LD (13.80 ± 0.03 µg GAE/g) and Ext-A-LR (15.21 ± 0.05 µg GAE/g), suggesting once again that the use of controlled temperature in the extraction of bioactive compounds from *P. cupana* is recommended, possibly because it increases the solubility of bioactive compounds in the plant matrix [39].

It is important to mention that some phytochemicals present in *P. cupana* may not be extracted and identified when using water as the sole extraction solvent, given their low polarity or insolubility in aqueous media, such as phenolic compounds that have an aromatic structure and hydrophobic side chains [40,41]. However, in our study, this seems to have been partially overcome in view of the metabolites described in Figure 1, which demonstrate that the emphasis given to using only aqueous extractions was effective in noting compounds of different classes and eliminates the probable influences of organic solvents on the synthesis of AgNPs.

### 2.3. Optical Properties by UV-Vis Spectrophotometry

**Figure 2:**
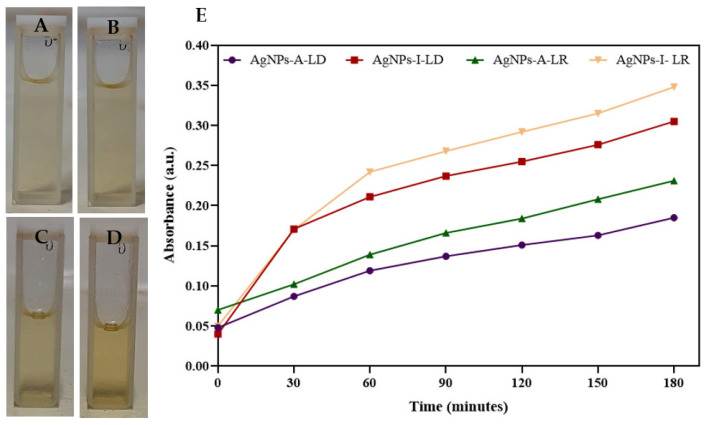
Visual aspects of AgNP colloidal suspensions—(**A**) AgNPs-A-LR: silver nanoparticles synthesized using leaf extract from the dry season prepared by agitation; (**B**) AgNPs-I-LR: silver nanoparticles synthesized using leaf extract from the dry season prepared by infusion; (**C**) AgNPs-A-LR: silver nanoparticles synthesized using leaf extract from the rainy season prepared by agitation; (**D**) AgNPs-I-LR: silver nanoparticles synthesized using leaf extract from the rainy season prepared by infusion. (**E**) Kinetics of AgNPs formation by UV/Vis analysis at 450 nm over 180 min.

The successful synthesis of AgNPs was initially monitored by the color change of the reaction mixture containing each extract prepared with *P. cupana* leaves collected at different seasonal periods and AgNO_3_, which at the end of the synthesis appeared in different shades of yellow, indicating the transformation of Ag^+^ ions to Ag^0^ (Figure 2A–D). This color change, which intensified during synthesis, is the result of the SPR (surface plasmon resonance) phenomenon, which is due to the collective oscillation of free electrons in the presence of visible light [42,43].

Given that AgNP suspensions exhibit a considerable absorption band in the visible spectrum, their formation and stabilization can be detected by optical properties due to color changes that can be confirmed with the naked eye and spectrophotometrically [44]. In our study, monitoring the absorbance of colloidal suspensions at a wavelength of 450 nm every 30 min of reaction shows that the nanostructures synthesized with leaf extracts prepared by infusion had higher intensity (0.305 and 0.348 a.u.) compared to those obtained from leaf extracts prepared by agitation (0.185 and 0.231 a.u.) (Figure 2E). This direct correlation between the method of extract preparation and AgNP formation may be related to the greater availability of metal ion-reducing metabolites that are extracted using controlled temperature, including polyphenols that control particle growth, prevent agglomeration, and allow the formation of nanostructures [45].

**Figure 3:**
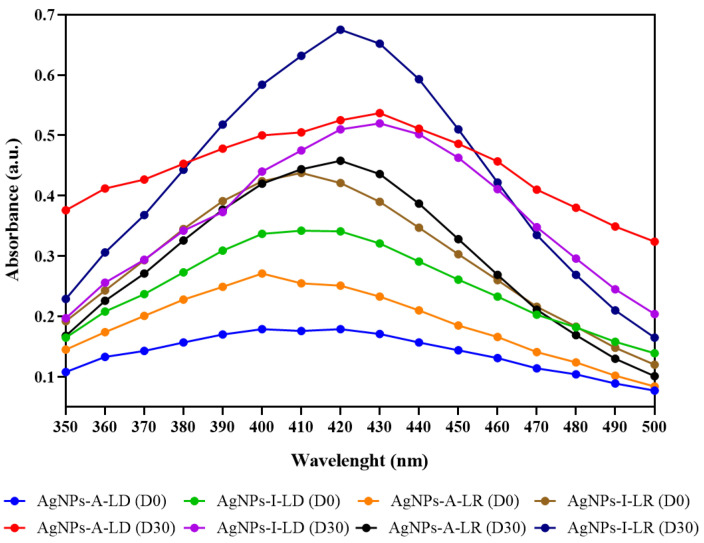
Absorption spectra of AgNPs on the day of synthesis (D0) and after 30 days (D30).

AgNPs exhibited absorption bands at different wavelengths depending on the sample/day of analysis (Figure 3), with these bands being influenced by several factors, including size, morphology, dielectric environment, and reducing agents [46,47]. In our study, all colloidal suspensions exhibited increased absorbance after one month of storage under refrigeration (4 °C) and protected from light, suggesting a continuous reduction in Ag^+^ ions and possible growth or reorganization of AgNPs over time. Specifically, AgNPs-A-LD showed an increase in absorbance from 0.179 a.u. on the day of synthesis to 0.537 a.u. after 30 days (~200%), while AgNPs-A-LR increased from 0.271 to 0.458 a.u. (~69%). Slightly different findings were seen in relation to the nanostructures of the rainy season, in which similar increases of around 52% (0.342 a.u. to 0.520 a.u.) and 54.1% (0.438 a.u. to 0.675 a.u.) were observed from day one to day thirty for AgNPs-I-LD and AgNPs-I-LR, respectively. This indicates that during the month of storage, the residual Ag+ ions continued to be reduced to AgNPs, increasing the absorbance intensity, which may affect the size and stability of the particles [48,49].

In addition, shifts in the wavelength of maximum absorption were observed throughout the monitoring period for AgNPs-I-LR (410 to 420 nm) and AgNPs -A-LD (420 to 430 nm), but larger deviations toward the red region in the UV/Vis spectrum were noted in AgNPs-A-LR (400 to 420 nm) and AgNPs-I-LD (410 to 430 nm). These data are consistent with previous reports that showed native species from the Amazon region being exploited in the sustainable synthesis of AgNPs with absorption between 410 and 430 nm from aqueous extracts of the leaves and fruits of *Eugenia stipitata*, *Mauritia flexuosa*, and *Oenocarpus bacaba* [50,51].

According to Mie theory, a single band in the UV/Vis spectrum suggests the presence of spherical nanostructures. In turn, this fact is reinforced by previous studies that showed that spherical AgNPs contribute to maximum absorption bands around 400 nm [52,53], which was observed in the present study during the spectrophotometric stability assessment. Furthermore, the broadening of these bands or even the appearance of two or more bands is due to the anisotropic nature of the particles and may indicate their polydispersity, representing a preliminary way to make inferences about the properties and long-term stability of AgNPs [54,55,56].

### 2.4. Stability Analyses by Dynamic Light Scattering (DLS) and Zeta Potential

The DLS technique measures the diameter of several particles simultaneously when dispersed in liquid media, and this measurement is determined by Brownian motion and the intrinsic properties of each sample after interacting with the used light scattering. Since the solvation layer on the surface of the particles is included in the measurements, the sizes determined by this technique tend to be larger than those obtained by other analyses that investigate the dry diameter [57,58].

**Figure 4:**
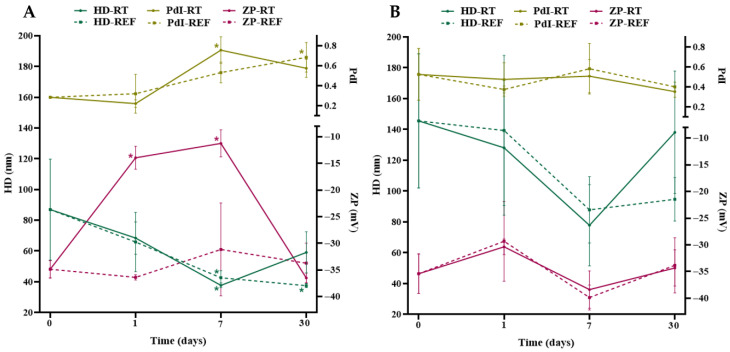
Analysis of the hydrodynamic diameter (HD), polydispersity index (PdI), and surface Zeta potential (ZP) of AgNPs synthesized from leaf extracts from the dry (**A**) and rainy (**B**) seasons prepared by agitation and kept under two storage conditions, room temperature (RT) and refrigeration (REF), for 30 days. The symbol “*” indicates statistically significant differences (*p* < 0.05) by the one-way ANOVA test followed by Tukey’s test in relation to the measurement on day 0.

According to Figure 4A and Supplementary Table S1, the evaluation of the colloidal characteristics of AgNPs-A-LD revealed significant decreases (*p* < 0.05) in the HD of the nanostructures after seven (37.67 ± 1.56 and 42.64 ± 4.54 nm) and thirty days (37.49 ± 1.37 nm), in both storage conditions, when compared to the initial measurement, which was 86.97 ± 32.82 nm. Significant variations (*p* < 0.05) were also observed in the PDI, with high values (0.684–0.755) when compared to the initial value of 0.284 ± 0.01, reflecting the polydispersity of the particles in aqueous medium and the likely impact on their biological activities [59]. Regarding ZP, the value obtained for the newly synthesized AgNPs was −34.9 ± 1.65 mV and decreased significantly (*p* < 0.05) to −14 ± 2.15 and −11.3 ± 2.54 mV after one and seven days, respectively, at room temperature.

In turn, as described in Figure 4B and Supplementary Table S1, AgNPs -A-LR AgNPs presented HD above 100 nm, except for the aliquots analyzed after seven days (77.78 ± 26.44 and 87.82 ± 21.56 nm) and the one that remained in the refrigerator for thirty days (94.69 ± 14.14 nm); these suspensions stood out as the samples with the highest mean deviations, which may result in intermediate polydispersity. This was indeed observed, since the PdI values were considered moderate to high (0.354 to 0.583), not differing significantly from the initial value, which was 0.526 ± 0.259. On the other hand, the ZP values indicate adequate colloidal stability, in addition to good homogeneity conditions [60], considering that after seven days the values were above −33 mV, similar to the initial measurement of −35.4 ± 3.7 mV.

**Figure 5:**
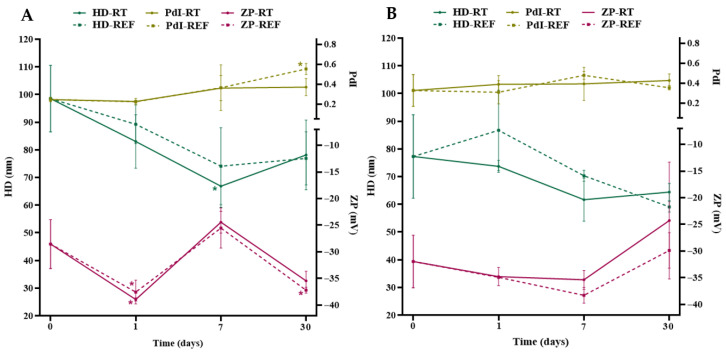
Analysis of the hydrodynamic diameter (HD), polydispersity index (PdI), and surface Zeta potential (ZP) of AgNPs synthesized from leaf extracts from the dry (**A**) and rainy (**B**) seasons prepared by infusion and kept under two storage conditions, room temperature (RT) and refrigeration (REF), for 30 days. The symbol “*” indicates statistically significant differences (*p* < 0.05) by the one-way ANOVA test followed by Tukey’s test in relation to the measurement on day 0.

The results of the AgNPs-I-LD analyses (Figure 5A, and Supplementary Table S1) showed that there was a lower tendency for changes in the colloidal properties of the nanostructures, since only after seven days stored at room temperature (66.89 ± 7.64 nm) and after thirty days in the refrigerator (0.552 ± 0.055) were significant differences (*p* < 0.05) in HD and PdI detected, respectively, in relation to the value obtained on the day of synthesis, which was 98.51 ± 12.04 nm and 0.246 ± 0.021. Furthermore, it was noted that the PZ values were between −35.4 ± 1.79 and −38.9 ± 0.32 mV in the analyses after one and thirty days, being significantly different (*p* < 0.05) from the initial value, which was −28.5 ± 4.6 mV, representing an improvement in long-term stability due to the repulsive electrostatic forces between the nanostructures [61].

As for the analyses for AgNPs-I-LR (Figure 5B, and Supplementary Table S1), it was found that HD underwent reductions from the first week of analysis, but without significant differences in relation to the value of the newly synthesized nanostructures, which was 77.28 ± 15.06 nm. Regarding PdI, it is possible to observe the same trend described above, in which values from 0.309 to 0.478 denote moderately polydispersed particle populations, suggesting a greater tendency to aggregation [62] and without significantly differing from the measurement obtained on the day of synthesis, which was 0.326 ± 0.158. In turn, the ZP averages remained above −30 mV until the first week of analysis but decreased in the thirty-day analyses (−24.3 to −29.9 mV), suggesting a probable loss of stability.

Our results appear to be the first record of the influence of the type of preparation of aqueous extracts of *P. cupana* and the seasonal time of collection of plant material on the physicochemical and colloidal characteristics of AgNPs. Associating the obtained results, we observed that there was a subtle tendency toward the formation of smaller and less homogeneous nanostructures after thirty days when using leaf extracts from the dry season prepared by agitation when compared to nanostructures prepared from leaf extracts from the rainy season. Additionally, in relation to AgNPs synthesized from leaf extracts prepared by infusion, we found that, even with the increase in polydispersity throughout the monitoring, the particle populations remained stable, given the ZP values. Thus, we reinforce the importance of using temperature during the extraction of bioactive compounds from the guarana plant, which, in turn, can act in the coating of AgNPs through adsorption on the surface of the particles, mainly due to the presence of biomolecules with -OH and -COOH groups [63].

Even though the reproducibility of the characteristics of nanomaterials synthesized by green synthesis routes is difficult to achieve, our results indicate an improvement in this scenario, given the possibility of understanding the synthesis process based on the variables that were investigated. In this sense, the extract from the pulp of the Amazonian plant *Euterpe oleracea* prepared at room temperature aided in the formation of AgNPs of up to 129.3 nm, with a PdI of 0.452 and surface charge of −37.2 mV [64]. Recently, the infusion method was used in the preparation of aqueous extracts from the seed coat of *P. cupana*, which, when used in the biosynthesis of AgNPs, resulted in particles with 63.9 nm, a PdI of 0.448, and ZP of −11.2 mV [28].

In this context, in relation to the different characteristics obtained for AgNPs from the dry and rainy seasons, biotic and abiotic factors, such as geographical and seasonal variations, can influence the presence/availability of bioreductive metabolites in plant tissues and compromise the success of biogenic syntheses [65,66,67]. Monitoring AgNP synthesis from *Pterodon emarginatus* extract showed that the diameter after one month when using leaf extract collected in winter was greater (107.3 nm) compared to those collected in summer (71.89 nm), with a PD of 0.3 and ZP below −29 mV [68]. The extract from the leaves of the medicinal plant *Dipteryx odorata* collected during the rainy season was used in the biosynthesis of AgNPs with an HD of 130.3 nm, PdI of 0.113, and ZP of −7.2 mV [69]. In our previous study, seasonal leaf extracts of guarana prepared by boiling were used in the biosynthesis of AgNPs with an average diameter between 74.28 and 101.6 nm, PdI between 0.299 and 0.479, and ZP between −16. 8 and −39.1 mV after thirty days of stability [22].

### 2.5. Nanoparticle Tracking Analysis (NTA)

The use of NTA to characterize and quantify the size and concentration of nanostructures offers significant advantages over traditional techniques, including the number of individual particles counted in real time and the analysis time required to obtain reliable size distributions [70].

In this study, the results indicated a modulation of the hydrodynamic diameter and Span index (SI) correlated with the form of preparation of the leaf extracts (Table 3). This was due to the smaller sizes and degrees of dispersity of AgNPs-I-LR and AgNPs-I-LD, with 39.4 ± 16.1 nm (SI 0.342) and 60.8 ± 0.9 nm (SI 0.591), respectively, with a mostly monomodal particle distribution, and without significant secondary populations (Supplementary Figure S2). In turn, the nanostructures synthesized with the extracts prepared by agitation, regardless of the collection season, had multimodal distributions with an SI of 0.863 and 1.014, in addition to averages of 96.6 ± 5.2 nm (AgNPs-A-LD) and 77.6 ± 2.1 nm (AgNPs-A-LR), respectively (Supplementary Figure S2). The performed analyses showed that the green synthesis of AgNPs using guarana leaf extracts resulted in colloidal suspensions containing 3.71 × 10^10^ particles/mL for AgNPs-A-LD and 3.89 × 10^9^ particles/mL for the AgNPs-I-LR sample, followed by 8.60 × 10^7^ particles/mL for AgNPs-A-LR and a slightly lower concentration in the AgNPs-I-LD suspension with 6.46 × 10^7^ particles/mL (Table 3).

Although there are variations between particle sizes using different techniques, in general, the values obtained by NTA correspond to the size range of nanostructures obtained by DLS, given the hydrodynamic environment common to both analyses, which is considered closer to what is expected in an in vivo system. In turn, knowing the concentration of AgNPs present in a sample can facilitate necessary and important adjustments for biological assays, standardizing the developed synthesis and characterization procedures.

In this sense, our findings are in line with previous research in which biogenic AgNPs from *Althaea officinalis* leaves had a diameter of 131 nm, with 7.2 × 10^10^ particles/mL [71], and, more recently, the concentration of AgNPs synthesized with extracts from *Citrus limon* peel was 4.22 × 10^10^ particles/mL and 60 nm in diameter [72]. In addition, some studies investigating freeze-dried or fresh leaf extracts from *Barleria albostellata*, *Paullinia cupana*, and *Tabernaemontana ventricosa* used in the green synthesis of AgNPs highlight colloidal suspensions with an average size of 53.9–57.9 nm, 68.5–89.3 nm, and 120.5–125 nm, respectively, obtained by NTA [23,73,74].

### 2.6. Morphological Analyses (TEM) and Elemental Composition (EDX)

**Figure 6:**
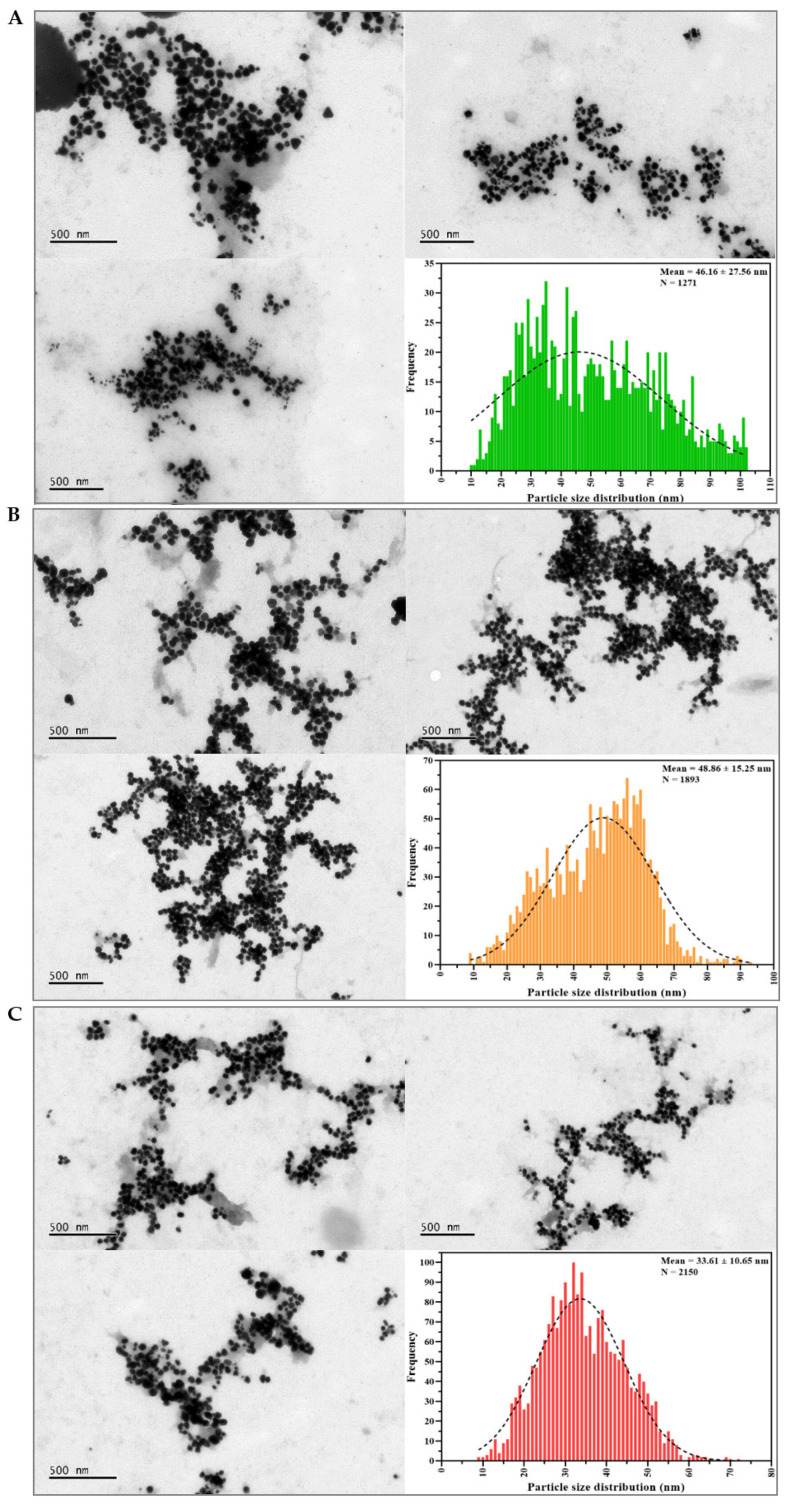

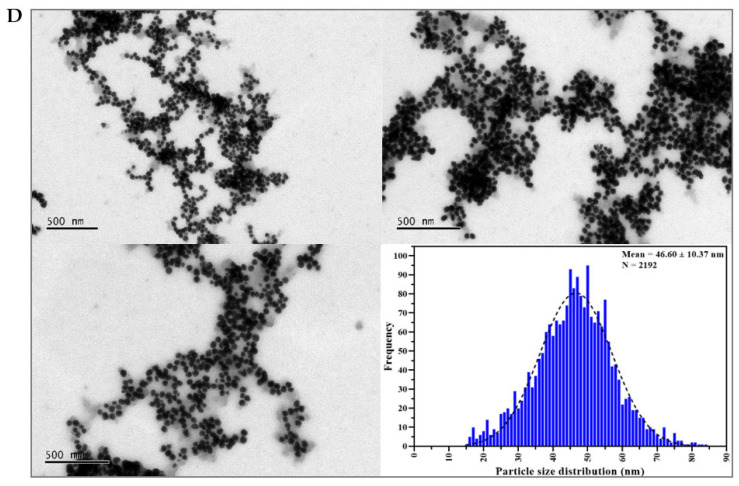
TEM images at 8.000 or 10.000× magnification and particle size distribution histograms of (**A**) AgNPs-A-LD, (**B**) AgNPs-I-LD, (**C**) AgNPs-A-LR, and (**D**) AgNPs-I-LR.

TEM analysis was used to evaluate the shape and diameter of AgNPs biosynthesized from seasonal aqueous leaf extracts of guarana, as shown in Figure 6. The results revealed that the nanostructures from the dry season had a wide particle size distribution compared to those from the rainy season, where the distribution was narrower. This may be influenced by the number of particles counted in each group, which was up to 1893 or above 2100, respectively. This may be related to the varied morphologies observed in each sample, in addition to signifying the efficiency in controlling the formation and growth of AgNPs.

In Figure 6A, the AgNPs-A-LD sample had particles ranging in size from 10.49 to 141.2 nm and an average of 46.16 ± 27.56 nm, like that found for AgNPs-I-LD with a distribution between 8.97 and 94.95 nm and an average size of 48.86 ± 15.25 nm (Figure 6B). In turn, the average diameter of AgNPs-A-LR was 33.61 ± 10.65 nm, with a distribution ranging from 8.66 to 90.06 nm (Figure 6C), while AgNPs-I-LR had a distribution between 15 and 91.83 nm, as well as an average size of 46.60 ± 10.37 nm (Figure 6D). These results corroborate other investigations that described nanoscale diameters for AgNPs derived from aqueous extracts of Amazonian plants, such as 19.4–20.7 nm when using *Euterpe oleracea* and 20–30 nm when using *Astrocaryum aculeatum* [75,76]. Additionally, for *Paullinia cupana*, the AgNP production route was investigated in our previous study and showed that by varying the energy source during synthesis, the diameter obtained by TEM varied between 13.08 ± 6.30 nm in an autoclave and 61.19 ± 19.10 nm in a water bath [77].

In general, the fields in which the images were captured show partial agglomerations of nanostructures that may have been caused by moderate PdI values or sample preparation but which did not compromise the observation/sharpness of their characteristics when dehydrated and analyzed by TEM. Another important highlight is the apparent shaded layer that accompanies and coats the surface of AgNPs, suggesting the presence of coverage by water-soluble biomolecules from the extract and demonstrating that colloidal dispersions can remain stable in solution [78].

As expected, according to the biological synthesis pathway, the morphology of AgNPs presented mainly spheroidal, quasi-spherical, triangular, hexagonal, rod-shaped, and prismatic shapes, regardless of the nanostructure group. It is important to note that the varied morphologies may be due to the phytochemical nature of the analyzed extracts, as well as the effect of seasonality, which favors the possibility of chelation/nucleation/reduction of metal ions in different ways depending on the variables involved in the synthetic process, as already described in previous studies [79].

**Figure 7:**
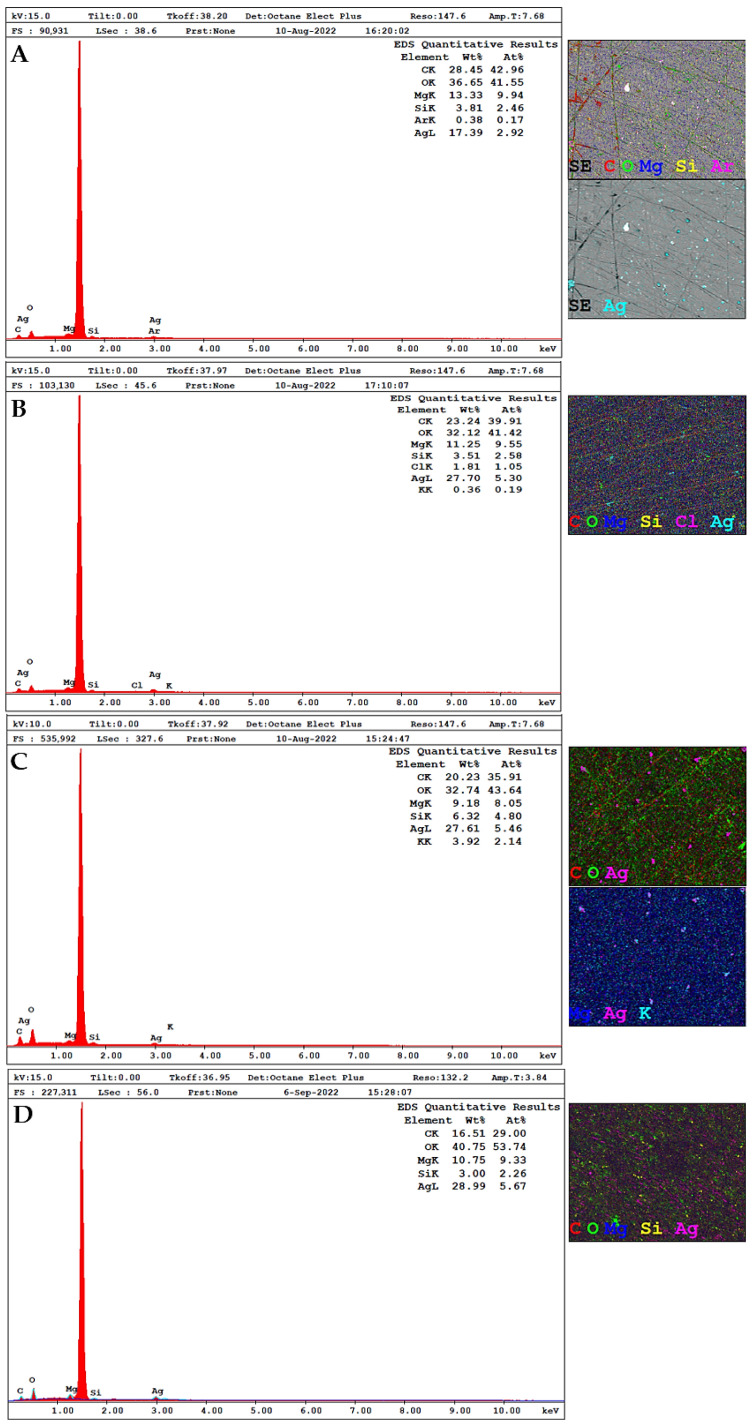
EDX spectra of AgNPs and elemental mapping of atoms present in each sample. (**A**) AgNPs-A-LD, (**B**) AgNPs-I-LD, (**C**) AgNPs-A-LR, and (**D**) AgNPs-I-LR.

The EDX technique was used to analyze the elemental composition and stoichiometry of the AgNPs (Figure 7). At around 3 keV, a signal characteristic of the absorption of metallic silver was generated, caused by the SPR effect, confirming the successful synthesis of the biogenic nanostructures [80]. The silver content, in mass percentage (%), showed a relative proportion of 17.39% (AgNPs-A-LD), 27.70% (AgNPs-I-LD), 27.61% (AgNPs-A-LR), and 28.99% (AgNPs-I-LR), as shown in Figure 7A–D, respectively, similar to that recorded in a previous study in which this content represented 27.34% in AgNPs synthesized from the extract of the medicinal plant *Cornus officinalis* [81]. Next to each spectrum are images of the elemental mapping, confirming the spatial and homogeneous distribution of silver.

Some other signals were attributed in the performed analyses and refer to the presence of different atoms in the samples, such as the high content of carbon (C) and oxygen (O), in addition to peaks indicative of magnesium (Mg), silicon (Si), argon (Ar), chlorine (Cl), and potassium (K). These elements probably originate from the biomolecules of plant extracts that are bound to the surface of the particles, confirming the influence of metabolites on the stabilization of AgNPs in suspension [73,82]. It is worth noting that the intense peak around 1.5 keV is due to aluminum (Al), which is the main component of the support on which the nanostructures were previously deposited for reading by the equipment.

## 3. Material and Methods

### 3.1. Chemicals and Reagents

In this study, all chemicals were used without any further purification. The formic acid (CH_2_O_2_) and acetonitrile (C_2_H_3_N) were of LC-MS analytical grade (>98%) and obtained from Sigma Aldrich (St. Louis, MO, USA), as well silver nitrate (AgNO_3_) and free radicals DPPH and ABTS that were from the same manufacturer. Methanol (CH_3_OH), gallic acid (C_7_H_6_O_5_), and Folin–Ciocalteu are from Dinâmica (Indaiatuba, São Paulo, Brazil).

### 3.2. Procedures for Collecting Plant Material and Preparing Aqueous Extracts from Paullinia cupana Leaves

The guarana leaves (*Paullinia cupana*) were collected from the same tree in the morning, during the dry season (August 2021) and rainy season (February 2022) on a private property in the city of Maués, state of Amazonas, Brazil (Sitio Putitanga 03°22′07″ S and 57°41′27″ W). All procedures for the collection, transport, and storage of plant tissues were authorized by the Genetic Heritage Management Council (CGEN) under number A5C4D66.

To prepare the aqueous extracts, the leaves, previously stored in a freezer at −80 °C, were thawed at room temperature and then washed with distilled water, neutral detergent, and again with plenty of distilled water to remove impurities from their surfaces. After drying the plant material using paper towels, approximately 1 g was cut into fragments of similar size (5 mm^2^) and the extracts were prepared by two methods consisting of (i) agitation—the plant parts were immersed in 10 mL of deionized water and kept in a magnetic stirrer (761–5, Fisatom, São Paulo, Brazil) for 30 min at 1000 rpm at room temperature—and (ii) infusion—the plant material was immersed in 10 mL of freshly heated deionized water for 1 min at 60 °C on a heating plate (RH Basic 2, IKA, Baden-Württemberg, Germany) and left to stand for 10 min, with the temperature controlled using a thermometer. Thus, the concentration of each aqueous extract was 0.1 g/mL (*m*/*v*), and they were used in biogenic synthesis immediately after preparation.

**Figure 8:**
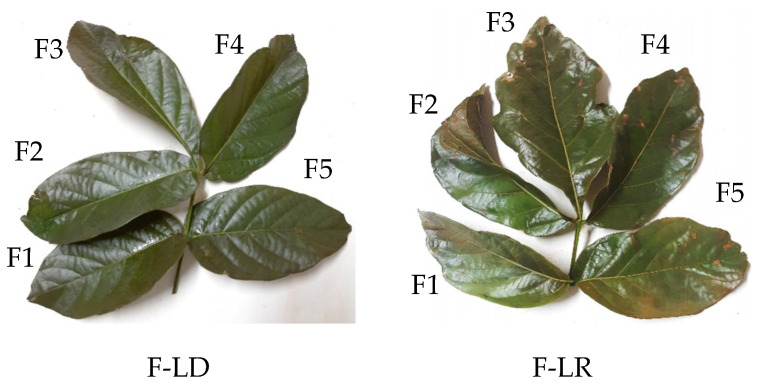
Guarana leaves (*Paullinia cupana*) collected during the two seasons of the year with leaflets, named F1, F2, F3, F4, and F5, identified in the same order, clockwise. F-LD: leaf collected during the dry season; F-LR: leaf collected during the rainy season.

Each extract was then filtered through qualitative filter paper (Whatman No. 1) and named Ext-A-LD or -LR (aqueous extract prepared by agitation with leaves collected during the dry or rainy season) and Ext-I-LD or -LR (aqueous extract prepared by infusion with leaves collected during the dry or rainy season). It should be noted that the guaranazeiro leaf is composed of five leaflets arranged in an orderly manner (Figure 8), and in this study, leaflet 2, collected during the dry season, was used in the preparation of extracts by the shaking and infusion methods, while leaflets 3 and 1, respectively, collected during the rainy season, were used in the preparation of extracts by these methods.

### 3.3. UHPLC-HRMS/MS Analyses of Seasonal Aqueous Leaf Extracts of Paullinia cupana

#### 3.3.1. Ultra-High-Performance Liquid Chromatography–High Resolution Mass Spectrometry/Mass Spectrometry (UHPLC-HRMS/MS)

The aqueous extracts from *P. cupana* leaves collected at different seasonal periods and prepared by the two methods were initially diluted to a concentration of 2 mg/mL in deionized water, centrifuged at 12,100 *g* for 10 min, and transferred to vial-type bottles. Chromatographic separations were performed using an ultra-high-performance liquid chromatography (UHPLC) system (Nexera X2, Shimadzu Corporation, Japan) consisting of an auto-vacuum degasser, quaternary gradient pump, autosampler, and column oven coupled to a high-resolution mass spectrometer (HRMS) using electrospray ionization and quadrupole time-of-flight technology (ESI-qTOF, maXis 4G, Bruker Daltonics, Bremen, Germany).

An Acquity UPLC HSST3 column (150 × 2.1 mm, 1.8 µm, Waters Technologies, Milford, MA, USA) was used, with mobile phase A containing 0.1% formic acid in water (*v*/*v*) and mobile phase B containing 0.1% formic acid in acetonitrile (*v*/*v*). The elution gradient was 0% B isocratic for 0–1 min, 0–25% B from 1–9 min, 25–50% B for 9–14 min, 50–100% B for 14–17 min, 100% B isocratic for 17–20 min, and 0% B for 20–25 min, linearly returning to the initial condition for equilibrium. All analyses were performed at a flow rate of 0.4 mL/min, injection volume of 1 µL, and column temperature of 40 °C. HRMS analyses, as well as automatic MS/MS acquisition, followed those previously described in a previous study [83]. In summary, the instrumental settings were positive mode with the electrospray source operating at 3.6 kV (ESI(+)-MS) and negative mode with the electrospray source operating at 3.8 kV (ESI(−)-MS). Nitrogen was used as the nebulization gas at a pressure of 4 bar, and the desolvation gas flow was set at 9 L/min at 200 °C.

UHPLC-HRMS/MS data were acquired using the oTofControl 3.4 and HyStar 3.2 applications, and the spectra obtained in each chromatogram were calibrated internally using sodium formate solution (5 mM) at the beginning of each chromatographic injection. Mass recalibration was performed using the equipment reference lists, in HPC mode, with a maximum standard deviation of 0.7 ppm. The first-order spectra (MS) were evaluated using the SmartFormula tool (DataAnalysis 4.2, Bruker Daltonics, Bremen, Germany) to predict the elemental formula of the detected ions calculated from the error values in ppm—based on the difference between the measured mass and the calculated mass—and mSigma values—based on the difference between the isotopic distribution pattern and the theoretical one. In addition, error values below 5 ppm and mSigma values below 20 indicate a good probability of accuracy in determining the identity of the compound, which was performed by Compound Crowler (DataAnalysis 4.2, Bruker Daltonics, Bremen, Germany) in the KEGG and METLIN databases. Second-order spectra (MS/MS) were studied using the MetFrag tool [84] based on the fragmentation profile of candidate molecules from different databases and their mass/charge (*m*/*z*) values, which are compared with those obtained in the spectra of the analyzed samples. A score is calculated using the fragments of the peaks, thereby obtaining information about the quality of the candidate spectrum assignment.

#### 3.3.2. Determination of Total Phenolic Content (TPC)

The aqueous extracts from *P. cupana* leaves were characterized for total phenolic content (TPC) following a previously adapted methodology for testing in 96-well microplates [85]. Briefly, 20 µL of each aqueous extract (100 mg/mL) prepared by shaking and infusion methods was mixed with 20 µL of 1 N Folin-Ciocalteau reagent, 20 µL of methanol, 180 µL of deionized water, and finally 60 µL of aqueous sodium carbonate solution (10%); this mixture was left to stand, incubated in the dark and at room temperature for 20 min. After this time, the phenolic content was measured by measuring the absorbance of the wells using a spectrophotometer coupled to a microplate reader (SpectraMax M3, Molecular Devices, São Paulo, Brazil) at 760 nm.

The standard curve was prepared using solutions of gallic acid (GA) in methanol at various concentrations, determined by the equation y= 0.01972x − 0.00204 (R^2^ = 0.9972), and the phenolic content was expressed as equivalents of the standard per mass of plant extract (µg GAE/g). Three independent experiments were performed, each in triplicate per standard/sample concentration, both for the standard curve levels and for the phenolic compound quantification tests.

#### 3.3.3. Antioxidant Capacity for Eliminating DPPH and ABTS Free Radicals

The antioxidant potential of aqueous extracts of *P. cupana* (100 mg/mL) prepared by different methods was evaluated by free radical scavenging in 96-well microplates. For DPPH, the assays were performed using 20 μL of each extract mixed with 280 μL of freshly prepared free radical solution (0.08 mM) in methanol; these mixtures were left to stand in the dark at room temperature for 30 min and then their absorbance was measured at 517 nm [86].

The cationic radical ABTS^+^ was prepared by incubating 2 mL of ABTS (7 mM) with 0.0352 mL of potassium persulfate (140 mM), both solubilized in deionized water, for 16 h in the dark at room temperature. After this time, the mixture was diluted in methanol until it reached an absorbance between 0.8 and 1 at 734 nm. Next, 20 µL of the extracts was mixed with 280 µL of ABTS^+^ methanolic solution and incubated for 20 min at room temperature and in the dark, with the absorbance of the wells subsequently read at 734 nm [87].

In these assays, a spectrophotometer coupled to a microplate reader (M3, Molecular Devices, São Paulo, Brazil) was used, and the calibration curves were constructed from different concentrations of gallic acid in methanol, represented by the equations y = −0.1138x + 0.5549 (R^2^ = 0.9946) for DPPH and y = −0.2422x + 0.4683 (R^2^ = 0.9937) for ABTS, with the results represented as equivalents of the standard per mass of plant extract (µg GAE/g). All tests were performed in three independent experiments in triplicate per standard/sample concentration, both for the curve levels and for the free radical scavenging potential tests.

### 3.4. Biogenic Synthesis of AgNPs

AgNPs were synthesized using an aqueous solution of silver nitrate (AgNO_3_) at 1 mM (approximately 170 µg/mL) and leaf extracts with volumes equivalent to a concentration of 1 mg/mL. To this end, 5.94 mL of the AgNO_3_ solution and 0.06 mL of each extract used as a source of metal ion-reducing metabolites were added to glass test tubes; immediately afterwards, the reaction mixtures were protected from light with aluminum foil to prevent photooxidation of the silver and evaporation of the liquid during synthesis. Incubation took place in a water bath (555, Fisatom, São Paulo, Brazil) closed with a lid for 180 min at 70 °C, and the resulting colloidal suspensions were named AgNPs -A-LD or -LR (silver nanoparticles synthesized from the extract prepared by agitation leaves collected in the dry or rainy season) and AgNPs-I-LD or -LR (silver nanoparticles synthesized from the extract prepared by infusing leaves collected in the dry or rainy season).

### 3.5. UV/Vis Spectrophotometry

The absorbance spectra were acquired using a UV1800PC spectrophotometer (Phenix, Blomberg, Germany). The synthesis reactions were monitored by measuring the absorbance of the samples (2 mL, without prior dilution) every 30 min at a wavelength of 450 nm. At the end of incubation in a water bath, the absorption curve of each sample was plotted in the range of 350 to 550 nm to investigate the bands with the highest intensity related to the synthesized nanostructures.

### 3.6. Dynamic Light Scattering (DLS) and Electrophoretic Mobility (Zeta Potential)

The hydrodynamic diameter (HD) and polydispersity index (PdI) were obtained by photon correlation spectroscopy, also called dynamic light scattering (DLS), while the surface zeta potential (PZ) was measured by electrophoretic mobility. These parameters were measured using a ZetaSizer Nano ZS (Malvern Instruments, Malvern, UK) with a He-Ne laser operating at 633 nm at an angle of 90°, an internal temperature of 25 °C, and a stabilization time of 120 s. The AgNPs were diluted 1:10 (*v*/*v*) in deionized water to a final volume of 1 mL, and three readings were taken for each sample, with each reading measured from 10 runs.

The colloidal stability of the nanostructures was investigated under different storage conditions: at room temperature (25 °C) and under refrigeration (4 °C), with analyses performed one, seven, and thirty days after the initial synthesis. In this study, these data were processed using ZetaSizer 7.13 software developed by the same manufacturer of the equipment.

### 3.7. Nanoparticle Tracking Analysis (NTA)

The hydrodynamic diameter (HD) and particle concentration were determined using a Nano-Sight NS300 device (Malvern Instruments, Worcestershire, UK) equipped with a 532 nm (green) diode laser. AgNPs were diluted at a ratio of 1:1000 (dry season samples) or 1:100 (rainy season samples) (*v*/*v*) in deionized water and injected with sterile syringes into the sample chamber [88], in quintuplicate. Videos of the particles moving from Brownian motion were captured and analyzed using NTA 3.2 software (Malvern Instruments, Malvern, UK).

To indicate how uniform the distribution of AgNPs sizes is, the Span index (SI) was evaluated, which represents the population dispersion of particles in suspension. For this purpose, Equation (1) was used based on the D10, D50, and D90 values, which are based on the diameter values previously obtained in the individual analysis of the suspended nanostructures.

(1) $$Span=(\frac{D90-D10}{D50})$$

### 3.8. Morphology (Transmission Electron Microscopy—TEM) and Elementary Composition (Energy-Dispersive X-ray—EDX) of AgNPs

The synthesized AgNPs were subjected to TEM analysis to investigate their morphology using a JEM-1011 transmission electron microscope (JEOL, Tokyo, Japan) operating at 80 kV. Approximately 5 µL of each colloidal suspension, without prior dilution, was deposited on a 400-mesh copper screen covered with Formvar^®^ film, which was kept protected from light at room temperature for 24 h for complete drying of the liquid. TEM images were acquired randomly, and for each group of AgNPs, the particles were counted using ImageJ software version 1.8.0 (National Institute of Health, Bethesda, MD, USA), and histograms of the dry diameter distribution of the nanostructures were plotted.

The elemental analysis of the atoms, as well as the determination of the silver content in the AgNPs, were performed using the EDX technique in a scanning electron microscope (FEI, INSPECT S50 with Everhart-Thornley detector, Hillsboro, OR, USA) at 10 kV. Approximately 10 µL of each sample, without dilution, was dripped onto aluminum supports (stubs) suitable for the equipment to be used; these stubs remained for three days in a closed container, protected from light and at room temperature for drying, and then the analyses were performed without metal coating.

### 3.9. Statistical Analysis

The values referring to the characterization analyses of the extracts (TPC, DPPH, and ABTS), as well as the physicochemical and morphological characterizations obtained by DLS, electrophoretic mobility, NTA, and TEM are presented as mean ± standard deviation (SD) of the mean. Graphs and histograms were plotted using GraphPrism 8 software (GraphPad Software, San Diego, CA, USA).

## 4. Conclusions

In this study, we present an eco-friendly, simple, and rapid method for the synthesis of AgNPs from the aqueous extract of *Paullinia cupana* leaves, which has the potential to be used with a variety of plant extracts. Our approach was efficient for the development of nanostructures with multifunctional properties, minimizing the use of toxic chemicals and replacing them with natural sources native to Brazilian biodiversity that acted as reducing agents, stabilizers, and coatings for nanoscale particles.

When evaluating the combination of the influence of seasonality and the methods of preparation of the extracts on the final characteristics of the AgNPs, we observed variations in the spectral, dimensional, electrical, compositional, and morphological data that suggest a probable modulation in relation to the optimal conditions to produce these nanostructures to test them for their bioactivities. These findings obtained from the variables described above are still considered scarce in scientific publications, but they reinforce the need for in-depth investigations into the effects that such parameters have on the biogenic AgNPs formed in studies involving biological matrices and nanotechnology.

## Figures and Tables

**Table 1:** Characterization of biomolecules identified by UHPLC-HRMS/MS in positive (ESI+) and negative (ESI-) ionization modes in aqueous extracts of *Paullinia cupana* leaves collected during the dry season and prepared by agitation (Ext-A-LD) and infusion (Ext-I-LD), as well as those collected in the rainy season and prepared by agitation (Ext-A-LR) and infusion (Ext-I-LR).

POSITIVE MODE (+)					
**Ext-A-LD**					
**Peak**	**T_R_ (min)**	***m*/*z* [M + H]^+^**	**Molecular Formula**	**Compound Assigned**	**Class**
3	1.023	176.0915	C_7_H_13_NO_4_	Calistegin B2	Nortropane alkaloid
4	1.112	148.0973	C_6_H_13_NO_3_	Fagomine	Piperidine alkaloid
8	8.697	181.0722	C_7_H_8_N_4_O_2_	Theobromine	Methyl xanthine alkaloid
**Ext-A-LR**					
4	0.987	176.0915	C_7_H_13_NO_4_	Calistegin B2	Nortropane alkaloid
5	1.077	148.0970	C_6_H_13_NO_3_	Fagomine	Piperidine alkaloid
8	7.953	181.0712	C_7_H_8_N_4_O_2_	Theobromine	Methyl xanthine alkaloid
9	11.145	595.1652	C_27_H_30_O_15_	Flavone or flavonol derivative	Flavonoid glycosides
**Ext-I-LD**					
3	0.986	176.0917	C_7_H_13_NO_4_	Calistegin B2	Nortropane alkaloid
7	1.548	130.0860	C_6_H_11_NO_2_	Pipecolic acid	Carboxylic acid from piperidine
9	8.155	181.0722	C_7_H_8_N_4_O_2_	Theobromine	Methyl xanthine alkaloid
11	11.774	595.1667	C_27_H_30_O_15_	Saponarin	Flavonoid glycosides
12	12.155	865.1989	C_45_H_36_O_18_	Cinnamtannin D1	Proanthocyanidin
13	14.525	451.1241	C_21_H_22_O_11_	Astilbin	Flavonoid glycosides
13	14.525	305.0656	C_15_H_12_O_7_	Taxifolin	Dihyroflavonols
14	15.096	303.0501	C_15_H_10_O_7_	Flavone or flavonol derivative	Flavones/Flavonols
**Ext-I-LR**					
5	0.994	176.0916	C_7_H_13_NO_4_	Calistegin B2	Nortropane alkaloid
6	1.072	148.0973	C_6_H_13_NO_3_	Fagomine	Piperidine alkaloid
13	11.159	291.0846 595.1638	C_15_H_14_O_6_	Flavone or flavonol derivative	Flavanols
14	11.503	865.1946	C_45_H_36_O_18_	Cinnamtannin D1	Proanthocyanidin
**NEGATIVE MODE (−)**					
**Peak**	**T_R_ (min)**	***m*/*z* [M + H]^−^**	**Molecular Formula**	**Compound**	**Class**
**Ext-A-LR**					
7	13.496	449.1074	C_21_H_22_O_11_	Astilbin	Flavonoid glycosides
8	14.320	447.0917	C_21_H_20_O_11_	Quercitrin	Flavonoid glycosides
10	15.262	431.0967	C_21_H_20_O_10_	Afzelin	Flavonoid glycosides
**Ext-I-LD**					
7	1.230	191.0563	C_7_H_12_O_6_	Quinic acid	Cyclic polyol
10	14.289	449.1104	C_21_H_22_O_11_	Astilbin	Flavonoid glycosides
12	14.870	447.0939	C_21_H_20_O_11_	Quercitrin	Flavonoid glycosides
**Ext-I-LR**					
9	11.101	289.0713	C_15_H_14_O_6_	Flavone or flavonol derivative	Flavanols
12	13.529	449.1097	C_21_H_22_O_11_	Astilbin	Flavonoid glycosides
14	14.097	449.1087	C_21_H_22_O_11_	Chalcone derivative	Chalcones
15	14.333	447.0936	C_21_H_20_O_11_	Quercitrin	Flavonoid glycosides
17	15.266	431.0983	C_21_H_20_O_10_	Afzelin	Flavonoid glycosides

**Table 2:** Total phenolic content and antioxidant potential against DPPH and ABTS free radicals of seasonal leaf extracts from *Paullinia cupana*.

Samples of Extracts	TPC (µg GAE/g)	DPPH (µg GAE/g)	ABTS (µg GAE/g)
Ext-A-LD	120.50 ± 0.04 ^d^	23.43 ± 0.06 ^b^	13.80 ± 0.03 ^b^
Ext-I-LD	203.50 ± 0.06 ^c^	34.38 ± 0.05 ^a^	17.60 ± 0.03 ^a^
Ext-A-LR	237.00 ± 0.05 ^b^	29.48 ± 0.04 ^ab^	15.21 ± 0.05 ^b^
Ext-I-LR	281.40 ± 0.04 ^a^	31.11 ± 0.04 ^ab^	17.75 ± 0.02 ^a^

Values are represented as mean ± standard deviation of triplicate experiments. Statistical analysis: One-way ANOVA test (*p* < 0.05), followed by Tukey’s test. Superscript letters indicate significant differences among samples of seasonal aqueous leaf extracts of *Paullinia cupana*. µg GAE/g: equivalent microgram of gallic acid per gram of sample.

**Table 3:** Hydrodynamic diameter values, particle concentration, and Span index obtained for AgNPs after NTA analysis.

Samples	Diameter (nm)	Concentration (Particles/mL)	*Span* Index
AgNPs-A-LD	96.6 ± 5.2	3.71 × 10^10^	0.863
AgNPs-I-LD	60.8 ± 0.9	6.46 × 10^7^	0.591
AgNPs-A-LR	77.6 ± 2.1	8.60 × 10^7^	1.014
AgNPs-I-LR	39.4 ± 16.1	3.89 × 10^9^	0.342

The values are represented as the mean ± standard deviation of the mean of triplicate measurements.

## Data Availability

The original contributions presented in this study are included in the article/Supplementary Materials. Further inquiries can be directed to the corresponding author.

## Supplementary Materials

The following supporting information can be downloaded at: https://www.mdpi.com/article/10.3390/ph19010072/s1, Figure S1: Chromatograms obtained by UHPLC-HRMS/MS of the aqueous extract of leaves of *Paullinia cupana*. (A) Positive ionization mode (ESI+); (B) Negative ionization mode (ESI–); Table S1: Monitoring of the colloidal stability of AgNPs using Dynamic Light Scattering (DLS) and surface Zeta potential for 30 days under two storage conditions: room temperature (RT) and refrigeration (REF); Figure S2: Size distribution histograms of (A) AgNPs-A-LD, (B) AgNPs-I-LD, (C) AgNPs-A-LR, and (D) AgNPs-I-LR obtained by NTA.
