# Supplementary material for: Biosynthesis of Silver Nanoparticles from Paullinia cupana Kunth Leaf: Effect of Seasonality and Preparation Method of Aqueous Extracts
Source: Pharmaceuticals (Basel). 2025 Dec 30;19(1):72. doi: 10.3390/ph19010072 (PMC12845482; doi:10.3390/ph19010072)
Supplement: Supplementary file 1 [file pharmaceuticals-19-00072-s001.zip › pharmaceuticals-4043643-supplementary.pdf]

Supporting Information

Biosynthesis of Silver Nanoparticles from *Paullinia cupana* Kunth Leaf: Effect of Seasonality and Preparation Method of Aqueous Extracts

Alan Kelbis Oliveira Lima [1,2,*], Tainá Pereira da Silva Oliveira [3], Isadora Florêncio [4], Alberto Gomes Tavares Junior [5], Victor Hugo Sousa Araújo [5], Arthur Abinader Vasconcelos [6,7], Marlus Chorilli [5], Hugo de Campos Braga [8], Dayane Batista Tada [8], Gerson Nakazato [9], Sônia Nair Báo [4], Paulo Sérgio Taube [10], José Antônio de Aquino Ribeiro [1], Clenilson Martins Rodrigues [1] and Mônica Pereira Garcia [2]

[1] Brazilian Agricultural Research Corporation (EMBRAPA), Embrapa Agroenergy, Brasília 70770-901, DF, Brazil
[2] Nanobiotechnology Laboratory, Institute of Biological Sciences, University of Brasília (UnB), Brasilia 70910-900, DF, Brazil
[3] Department of Genetics and Morphology, Institute of Biological Sciences, Darcy Ribeiro University Campus, University of Brasilia (UnB), Brasília 70910-900, DF, Brazil
[4] Microscopy and Microanalysis Laboratory, Department of Cell Biology, Institute of Biological Sciences, University of Brasília (UnB), Brasília 70910-900, DF, Brazil
[5] School of Pharmaceutical Sciences, São Paulo State University (UNESP), Araraquara 14800-901, SP, Brazil
[6] Structured Nanomaterials Study Group, Federal University of Western Pará (UFOPA), Santarém 68005-120, PA, Brazil
[7] Laboratory of Oils of the Amazon, Institute of Biological Sciences, Federal University of Pará (UFPA), Belém 66075-110, PA, Brazil
[8] Institute of Science and Technology, Federal University of São Paulo (UNIFESP), São José dos Campos 12231-280, SP, Brazil
[9] Basic and Applied Bacteriology Laboratory, State University of Londrina (UEL), Londrina 86057-970, PR, Brazil
[10] Institute of Biodiversity and Forests, Federal University of Western Pará (UFOPA), Santarém 68005-100, PA, Brazil
* Correspondence: kelbislima@gmail.com; Tel.: +55-93-99151-2101

Supplementary Figure S1: Chromatograms obtained by UHPLC-HRMS/MS of the aqueous extract of leaves of *Paullinia cupana*. A) Positive ionization mode (ESI+); B) Negative ionization mode (ESI–).

Ext-A-LD: Aqueous extract prepared by agitation leaves collected during the dry season; Ext-I-LD: Aqueous extract prepared by infusing leaves collected during the dry season; Ext-A-LR: Aqueous extract prepared by agitation leaves collected during the rainy season; Ext-I-LR: Aqueous extract prepared by infusing leaves collected during the rainy season.

A

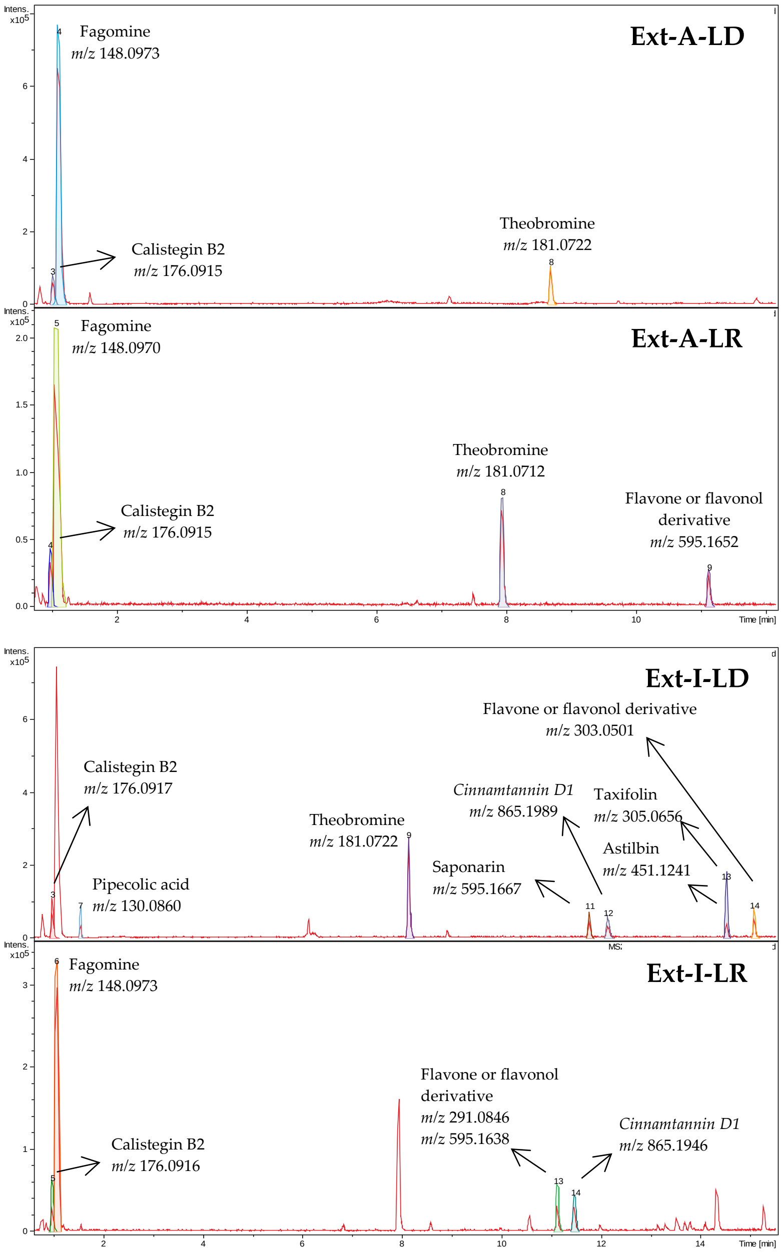

B

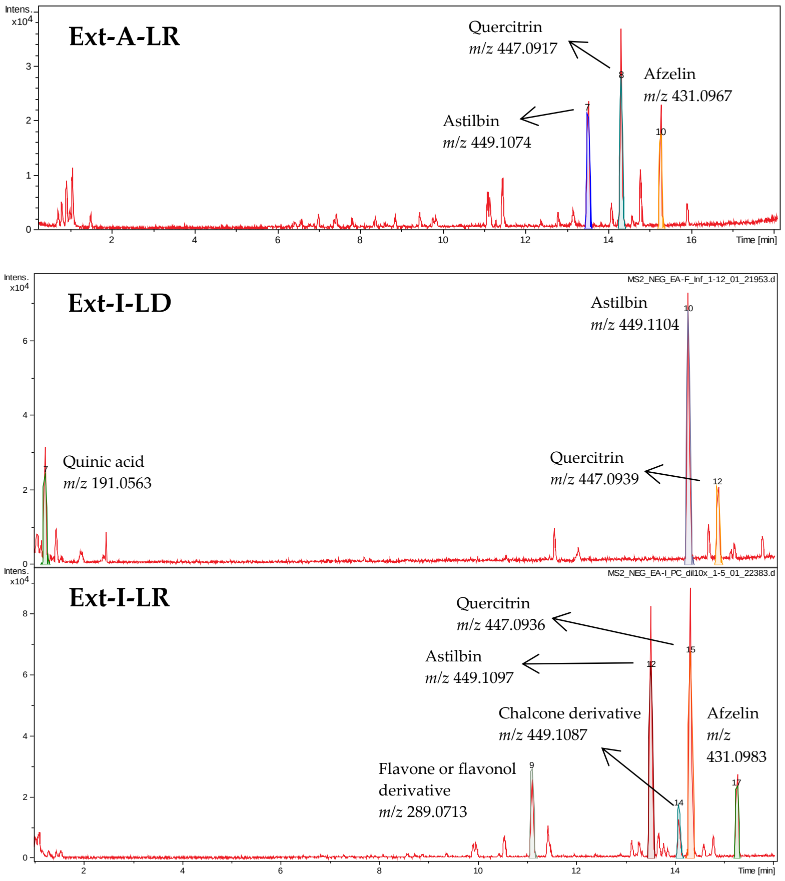
Ext-A-LR
Quercitrin
m/z 447.0917
Afzelin
m/z 431.0967
Astilbin
m/z 449.1074
Intens.
x10^4
Time [min]
Ext-I-LD
MS2_NEG_EA-F_Inf_1-12_01_21953.d
Astilbin
m/z 449.1104
Quinic acid
m/z 191.0563
Quercitrin
m/z 447.0939
Ext-I-LR
MS2_NEG_EA-I_PC_dil10x_1-5_01_22383.d
Quercitrin
m/z 447.0936
Astilbin
m/z 449.1097
Chalcone derivative
m/z 449.1087
Afzelin
m/z
431.0983
Flavone or flavonol
derivative
m/z 289.0713

Supplementary Table S1: Monitoring of the colloidal stability of AgNPs using Dynamic Light Scattering (DLS) and surface Zeta potential for 30 days under two storage conditions: room temperature (RT) and refrigeration (REF).

Time/Storage	AgNPs-A-LD		
	**HD (nm)**	**PdI**	**ZP (mV)**
D0	86.97 ± 32.82	0.284 ± 0.010	-34.9 ± 1.65
D1 – RT	68.48 ± 10.54	0.222 ± 0.037	-14.0 ± 2.15 *
D1 – REF	65.85 ± 19.26	0.321 ± 0.194	-36.4 ± 0.50
D7 – RT	37.67 ± 1.56 *	0.755 ± 0.133 *	-11.3 ± 2.54 *
D7 – REF	42.64 ± 4.54 *	0.532 ± 0.104	-31.2 ± 8.72
D30 – RT	59.07 ± 13.45	0.574 ± 0.090	-36.5 ± 0.92
D30 – REF	37.49 ± 1.37 *	0.684 ± 0.148 *	-33.7 ± 3.74

Time/Storage	AgNPs-A-LR		
	**HD (nm)**	**PdI**	**ZP (mV)**
D0	145.50 ± 43.52	0.526 ± 0.259	-35.4 ± 3.70
D1 – RT	128.10 ± 43.75	0.476 ± 0.167	-30.4 ± 1.46
D1 – REF	139.30 ± 48.72	0.376 ± 0.036	-29.3 ± 7.48
D7 – RT	77.78 ± 26.44	0.509 ± 0.167	-38.4 ± 3.50
D7 – REF	87.82 ± 21.56	0.583 ± 0.253	-39.9 ± 2.33
D30 – RT	138.10 ± 39.78	0.354 ± 0.056	-34.3 ± 3.39
D30 – REF	94.69 ± 14.14	0.401 ± 0.050	-33.8 ± 5.14

Time/Storage	AgNPs-I-LD		
	**HD (nm)**	**PdI**	**ZP (mV)**
D0	98.51 ± 12.04	0.246 ± 0.021	-28.5 ± 4.60
D1 – RT	83.03 ± 9.68	0.227 ± 0.030	-38.9 ± 0.32 ×
D1 – REF	89.25 ± 6.98	0.224 ± 0.015	-37.5 ± 2.25 ×
D7 – RT	66.89 ± 7.64 ×	0.361 ± 0.127	-24.4 ± 2.04
D7 – REF	74.11 ± 13.89	0.364 ± 0.230	-25.5 ± 3.77
D30 – RT	78.16 ± 12.57	0.370 ± 0.085	-35.4 ± 1.79
D30 – REF	76.94 ± 9.55	0.552 ± 0.055 ×	-37.2 ± 0.56 ×

Time/Storage	AgNPs-I-LR		
	**HD (nm)**	**PdI**	**ZP (mV)**
D0	77.28 ± 15.06	0.326 ± 0.158	-31.9 ± 4.91
D1 – RT	73.73 ± 2.16	0.388 ± 0.087	-34.8 ± 1.70
D1 – REF	86.73 ± 14.52	0.309 ± 0.117	-34.9 ± 0.17
D7 – RT	61.69 ± 7.77	0.393 ± 0.165	-35.4 ± 1.77
D7 – REF	70.32 ± 1.94	0.478 ± 0.038	-38.3 ± 1.47
D30 – RT	64.44 ± 3.14	0.426 ± 0.066	-24.3 ± 10.90
D30 – REF	59.10 ± 1.89	0.354 ± 0.022	-29.9 ± 3.32

Statistical analysis: One-way ANOVA test ($p<0.05$), followed by Tukey's test.

Values are represented as mean ± standard deviation (N = 3) of the mean of measurements in triplicate.

Superscript symbols indicate significant differences within each parameter separately for each AgNPs group compared to the day 0 (D0) measurement. *Represents the statistical differences for AgNPs-A-LD, ×Represents the statistical differences for AgNPs-I-LD.

Supplementary Figure S2: Size distribution histograms of (A) AgNPs-A-LD, (B) AgNPs-I-LD, (C) AgNPs-A-LR, and (D) AgNPs-I-LR obtained by NTA.

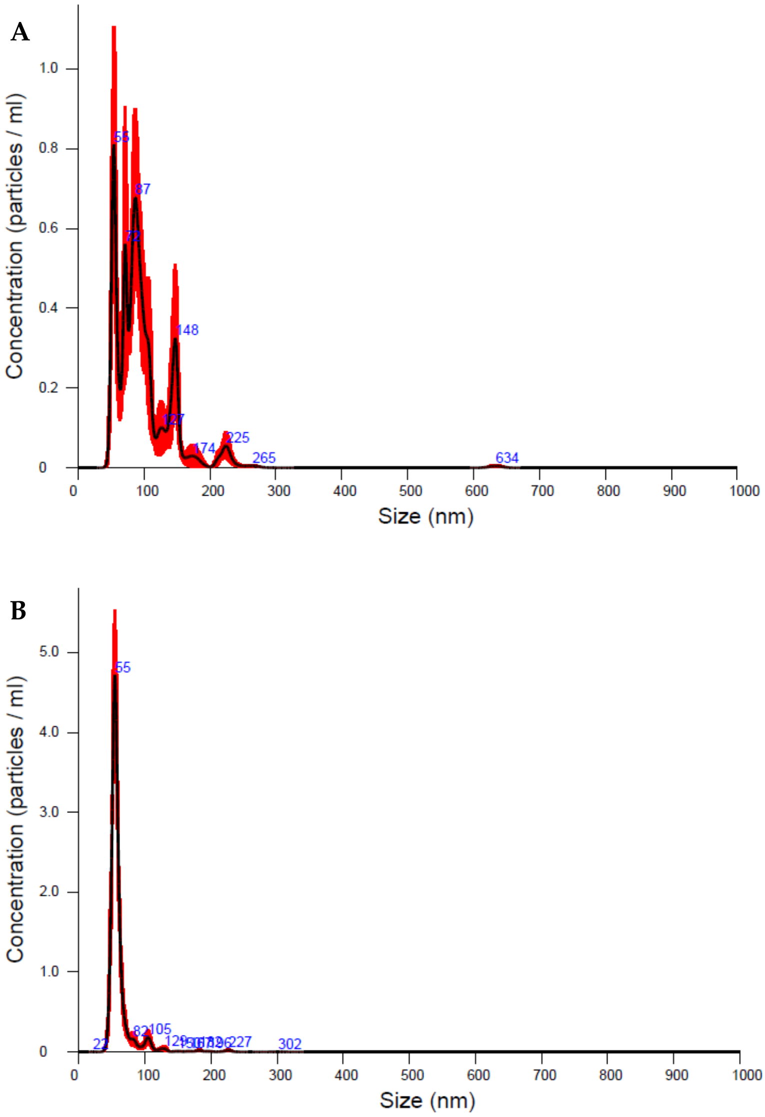

C

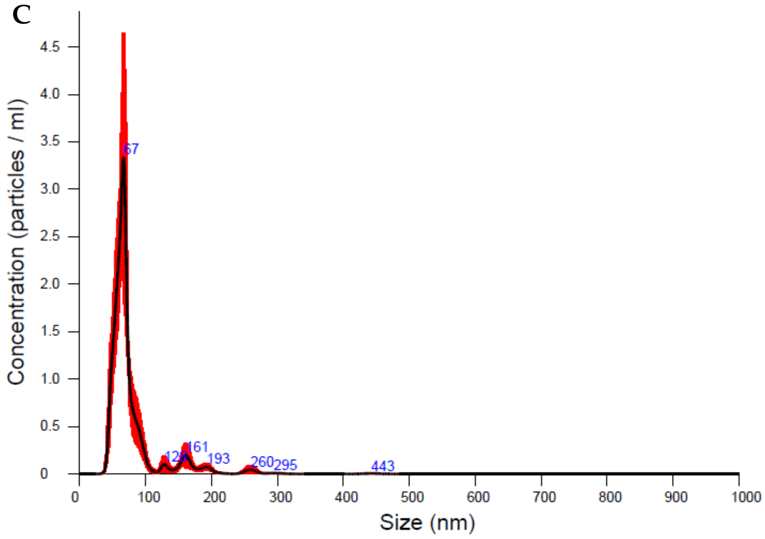

Concentration (particles / ml)
4.5
4.0
3.5
3.0
2.5
2.0
1.5
1.0
0.5
0
67
161
193
260
295
443
0
100
200
300
400
500
600
700
800
900
1000
Size (nm)

D

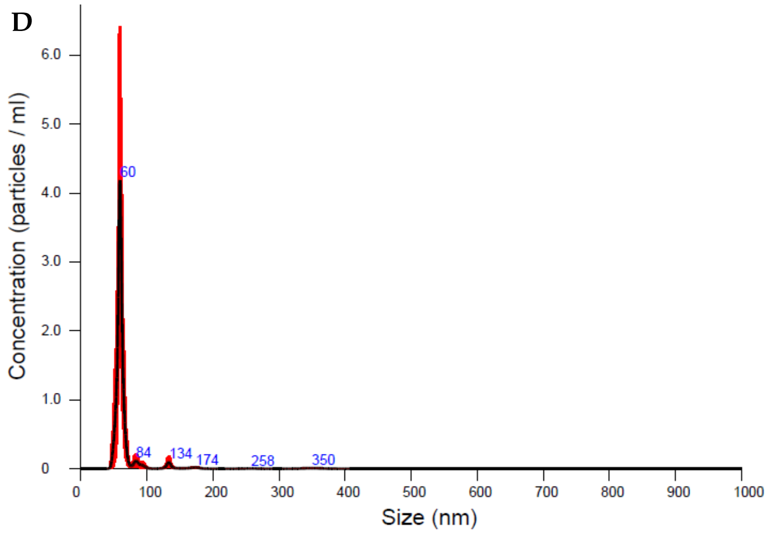

Concentration (particles / ml)
6.0
5.0
4.0
3.0
2.0
1.0
0
60
84
134
174
258
350
0
100
200
300
400
500
600
700
800
900
1000
Size (nm)
